# Irisin Regulating Skeletal Response to Endurance Exercise in Ovariectomized Mice by Promoting Akt/β-Catenin Pathway

**DOI:** 10.3389/fphys.2021.639066

**Published:** 2021-03-25

**Authors:** Renqing Zhao, Yalan Zhou, Jinqiao Li, Junjie Lin, Wei Cui, Yan Peng, Wenqian Bu

**Affiliations:** College of Physical Education, Yangzhou University, Yangzhou, China

**Keywords:** irisin, bone loss, exercise, ovariectomy, Akt/ß-catenin

## Abstract

**Purpose:** Thought irisin is recognized as a pivotal modulator for bone formation, its role in regulating skeletal response to exercise training remains unknown. Therefore, we aimed to determine the change of irisin in response to 8-week exercise training and its role in regulating the effects of exercise on bone loss in ovariectomized (Ovx) mice.

**Methods:** Forty 3-month old female C57BL/6 mic were randomly allocated into four groups: (1) Sham-operated (Sham); (2) ovariectomized; (3) Ovx plus 8-week downhill running exercise (Ex); (4) Ovx plus exercise and received twice weekly injection of cyclo RGDyk protein (a putative anti-irisin receptor agents) (ExRg).

**Results:** Ex group showed enhanced cortical and trabecular volumetric bone mineral density (vBMD) (*p* < 0.05), improved bone microarchitecture, and increased intensity of alkaline phosphatase positive (ALP^+^) cells compared with Ovx group. However, cyclo RGDyk administration weakened the exercise-related improvement of vBMD, BV/TV, and ALP intensity in bone. Serum estradiol, irisin, and bone alkaline phosphatase were higher, whereas circulating tartrate-resistant acid phosphatase was lower in Ex group compared with Ovx group (*p* < 0.05). Exercise promoted mRNA expression of fibronectin type III domain-containing protein 5 (FNDC5), Akt and β-catenin, and enhanced protein levels of FNDC5, the ratio of phosphorylated Akt (p-Akt) to Akt, and β-catenin (*p* < 0.05). When irisin pathways were blocked with cyclo RGDyk, increment of Akt, p-Akt/Akt, and β-catenin in Ex mice were attenuated.

**Conclusion:** It is suggested that irisin plays a potential role in regulating skeletal response to exercise partly through its interaction with Akt/β-catenin pathways.

## Introduction

Osteoporosis is a common systematic skeletal disease characterized by low bone mass and impaired bone geometry and microarchitecture, presenting a major health problem in older people worldwide (Van Den Bergh et al., 2012; Compston et al., 2019). Particularly, older females after menopause are often suffered from prolonged bone loss and increased fracture risk (Black et al., 2016). Therefore, effective strategies for prevention of osteoporosis is urgently needed for improving older people' health.

Exercise is known to increase muscle mass and strength (Rogers and Evans, 1993; Roig et al., 2008), promote bone mass accumulation (Zhao et al., 2014, 2015), and correct poor balance and posture control (Howe et al., 2011a,b), etc. Compelling evidence has proved exercise to be effective in preventing bone loss and subsequent fractures (Bonaiuti et al., 2002; Zhao et al., 2017a; Sherrington et al., 2019). Zhao et al. (2017b) reported that exercise training significantly increased bone density in postmenopausal women. Another study (Bonaiuti et al., 2002) demonstrated that exercise was effective in prevention of osteoporosis in older women. A Cochrane review (Sherrington et al., 2019) also reported exercise potentially reduced falls and subsequent fractures. However, the mechanism regulating the beneficial effects of exercise on bone is still unclear.

Irisin is a recently identified myokine which is secreted by muscle, increased with exercise, and produced by the cleavage of fibronectin type III domain-containing protein 5 (FNDC5) in humans and animals (Boström et al., 2012). It mediates certain favorable effects of exercise, especially showing to have positive effects on mechanical strain-related osteoblast differentiation (Qiao et al., 2016) and then preserve bone mass in ovariectomized (Ovx) mice (Colaianni et al., 2015). Recently, the discovery of the novel αV integrin receptors in bone cells further confirmed the likelihood that irisin plays a pivotal role in modulating interactions between exercise and bone formation (Kim et al., 2018). Osteoblast differentiation is mediated by several signaling pathways, and Akt/β-catenin pathway is recognized as an important modulator in osteoblastic differentiation in response to mechanical strain (Sunters et al., 2010). And recent studies reported that irisin regulated cell differentiation mainly through Akt/β-catenin pathway (Liu et al., 2015; Shi et al., 2017). Therefore, the current evidence indicates that irisin and Akt/β-catenin pathway play an important role in regulating osteoblastic differentiation. However, whether exercise intervention affects bone formation through irisin and its interaction with Akt/β-catenin pathway remains unclear. Therefore, we conducted 8-week exercise training in Ovx mice to determine the change of irisin in response to endurance exercise and its role in regulating the effects of exercise on bone loss.

## Materials and Methods

### Animals

The animals were cared for in accordance with *the principles of the Guide to the Care and Use of Experimental Animals*, and the protocol was approved by the Yangzhou University Institutional Animal Care and Use Committee (No. YZUDWLL-201905-001).

Forty 3-month old female C57BL/6 mice were purchased from Comparative Medical Center of Yangzhou University (Yangzhou, China). Four animals were housed per cage under the temperature of 23 ± 2°C and with a 12-h light-dark cycle. Food and drinking water were supplied *ad libitum*. One week after arrival, the mice were anesthetized, and sham-operated (Sham) or Ovx according to experimental protocols, and then two animals were housed for per cage. All mice were assigned randomly to 4 groups in parallel: (1) sham group; (2) Ovx group (Ovx); (3) exercise group (Ex): Ovx mice with exercise training for 8 weeks; (4) exercise and cyclo RGDyk treatment group (ExRg): Ovx mice received exercise and cyclo RGDyk [anti-irisin receptor agents (Kim et al., 2018)] interventions for 8 weeks.

### Intervention Protocols

Two weeks after operation, exercise mice received 1 week of adaptive training with a protocol of daily 30-min treadmill running and the speed gradually increasing from 8 meters/min to 13 meters/min (−0° to −8° grade). After adaptive training, exercise mice were trained regularly for 5 days per week, with each training section about 45 min at a speed of 13 meters/min and with the slope of −9°. After 3 weeks of surgical operation, ExRg group mice were treated twice weekly with 2.5 mg/kg cyclo RGDyk (GLPBIO Biological Company, USA) via tail vein injection (Kim et al., 2018). At the end of interventions, all mice were anesthetized and killed within 24 h. Blood and bone tissue samples were collected. Serum was separated by centrifugation at 5000 rpm for 20 min at 4°C and then kept at −20°C. Femora and tibia bone were dissected and stored in a freezer at −80°C until PCR and Western Blot analyses.

### μCT Analysis of Femur

The right femur was isolated and fixed in 4% paraformaldehyde and embedded in methyl methacrylate plastic after serial dehydration with a graded ethanol series to xylene. We used high-resolution desktop microcomputed tomography imaging (μCT40, Scanco Medical, Brüttisellen, Switzerland) for femur scanning. We assessed trabecular and cortical bone microstructure in the distal femur and femoral diaphysis. Image acquisition and analysis protocols adhered to the guidelines for the assessment of rodent bones by μCT (Bouxsein et al., 2010). In the distal femur, transverse μCT slices were evaluated in a region of interest beginning 0.2 mm superior to the distal growth plate and extending proximally 1.5 mm. The cortical and trabecular bone were identified for the following morphometric variables: bone volume fraction (BV/TV, %), volumetric bone mineral density (vBMD, g/cm^3^), trabecular thickness (Tb.Th, mm), trabecular number (Tb.N, 1/mm), and trabecular separation (Tb.Sp, mm), etc.

### Alkaline Phosphatase Staining and Quantitative Analysis

For ALP staining, proximal tibiae (left) were isolated and fixed in 10% paraformaldehyde fixation buffer (PFA), and decalcification performed with 10% EDTA. Eight μm-thick paraffin-embedded sections were obtained, and Improved Gomori calcium cobalt method was used to determine ALP activity after incubation with staining agents (Beijing Solarbio Science & Technology Co. Ltd, China). The slices were photographed under microscope (400× magnification). The images were analyzed using Image-Pro Plus image analysis software version 6.0 (IPP 6.0, Media Cybernetics, USA), and the areas and integral optical density (IOD) of ALP staining were quantified.

### Analysis of Serum Hormones and Biomarkers

The serum concentrations of estradiol (E_2_), irisin, bone alkaline phosphatase (BAP), and tartrate-resistant acid phosphatase (TRAP) were determined with ELISA kits (Jianglai Biological Company, Shanghai, China), according to the instructions in the manufacturer's protocol.

### Western Blot Analysis

Total protein (right tibia) was obtained using radioimmunoprecipitation assay (RIPA) lysis buffer (ApllyGen, Beijing, China). Protein concentration was determined using the BCA Protein Assay Kit (CWBIO, Beijing, China). Extracts were fractionated by SDS-PAGE and subsequently transferred to a polyvinylidene fluoride membrane (PVDF, IPVH00010, Millipore). After blocking with 3% non-fat dry milk in Tris-buffered saline (TBS), we incubated the membranes overnight at 4°C with antibody to FNDC5 (Abcam, Shanghai, China), β-catenin (Abcam, Shanghai, China), Akt (Cell Signaling Technologies, Hitchin, UK), and phospho-Akt (Ser473) (Cell Signaling Technologies, Hitchin, UK). For loading control, we used antibodies to β-actin. The secondary antibody was diluted to 1:2,000 and incubated with the membrane for 2 h at room temperature. After the last washing step, configure the luminescent liquid, soak the PVDF film with the luminescent liquid, and place it in the sample placement area of the ultra-high-sensitivity chemiluminescence imaging system (Chemi Doc™ XRS+, Shanghai, China) to run the program to develop the imaging.

### RT-PCR Analysis

Total RNA from right tibia was extracted using Trizol reagent (CWBIO, Beijing, China) according to the manufacturer's instructions. 200 ng of total RNA was reverse-transcribed with Ultrapure RNA extraction kit (CWBIO, Beijing, China) in a 20-μl cDNA reaction, as specified by the manufacturer. For quantitative PCR, the template cDNA was added to a 25 μl reaction with SYBR Green PCR Master Mix (Lifeint, Beijing, China) and 0.2 μM of primer. The amplification was carried out for 40 cycles under the following conditions: an initial denaturation of 95°C for 10 min, plus 40 cycles of 95°C for 10 s, then 60°C for 1 min. The fold changes were calculated relative to β-actin using the ΔΔ Ct method for FNDC5, β-catenin, and Akt mRNA analysis. The following primer sets were used: FNDC5: forward, AAGTGGTCATTGGCTTTGC; reverse, GTTGTTATTGGGCTCGTGT; β-catenin: forward, CACATCAGGACACCCAACGG; reverse, CGTATGTTGCCACGCCTT; Akt: forward, AAGCACCGTGTGACCATGAA; reverse, TTCTCAGTAAGCGTGTGGGC; β-actin: forward, AGGGAAATCGTGCGTGAC; reverse, CATACCCAAGAAGGAAGGCT.

### Statistical Analysis

Statistical analysis was performed using STATA software (*Version 15, StataCorp LP, Texas, USA*). Data were reported as mean and standard deviation (SD). A one-way analysis of variance (ANOVA) with the Bonferroni *post hoc* test was used for between-group comparisons. A level of *p* < 0.05 was accepted as significant.

## Results

### Changes in Weights and Serum Biomarkers

The baseline weights of mice were not different between groups (Table 1). At the end of 8-week interventions, the weights of Ovx mice significantly increased compared with Sham mice, but 8-week exercise training decreased the ovariectomy-induced increment of weights. The weights of ExRg group tended to decrease compared with that in Ovx group, but the difference was not significant. Ovariectomy markedly decreased uterus weight compared with Sham operation; exercise interventions seemed to improve the reduced uterus weight, but it was not significant (Table 1).

**Table 1:** Physical and serum parameters of mice between groups.

	**Sham (*n* = 10)**	**Ovx (*n* = 10)**	**Ex (*n* = 10)**	**ExRg (*n* = 10)**
Weight_pre_ (g)	20.96 ± 1.89	21.55 ± 2.06	21.21 ± 1.43	21.5 ± 2.55
Weight_post_ (g)	23.81 ± 2.48	27.99 ± 2.59^*^	24.13 ± 1.53^†^	25.29 ± 2.91
Uterus weigh (g)	0.069 ± 0.023	0.020 ± 0.012^*^	0.030 ± 0.017^*^	0.025 ± 0.018^*^
Serum E_2_ (pg/ml)	8.09 ± 2.47	4.05 ± 1.69^*^	7.13 ± 1.91^†^	3.97 ± 1.11^*^^§^
Serum irisin (pg/ml)	5.10 ± 1.96	2.04 ± 0.64^*^	4.34 ± 2.01^†^	3.05 ± 1.09^*^
Serum BAP (pg/ml)	0.56 ± 0.10	0.34 ± 0.10^*^	0.55 ± 0.07^†^	0.35 ± 0.12^*^^§^
Serum TRAP (pg/ml)	4.25 ± 0.61	6.01 ± 1.20^*^	4.02 ± 1.2^†^	5.22 ± 0.89^*^^§^

*Ovx, ovariectomized; Ex, exercise; ExRg, exercise plus cRGDyk; BAP, bone alkaline phosphatase; TRAP, tartrate-resistant acid phosphatase. Data are presented as mean ± SD*.

* *Denotes the difference between Sham group and Ovx, Ex, and ExRg groups was significant (p < 0.05)*;

† * indicates the difference between Ovx group and Ex and ExRg groups was significant (p < 0.05)*;

§ * presents the difference between Ex group and ExRg group was significant (p < 0.05)*.

Ovariectomy induced a marked reduction of serum levels of E_2_, irisin, and BAP, and up-regulated serum TRAP concentrations; the ovariectomy-induced changes of serum hormones and biomarkers were reversed by exercise interventions (Table 1). However, the increment of serum E_2_, irisin and BAP, and reduction of TRAP in Ex group were attenuated by cyclo RGDyk treatment (Table 1).

### Changes in Volumetric Bone Density and Bone Morphometry

**Figure 1:**
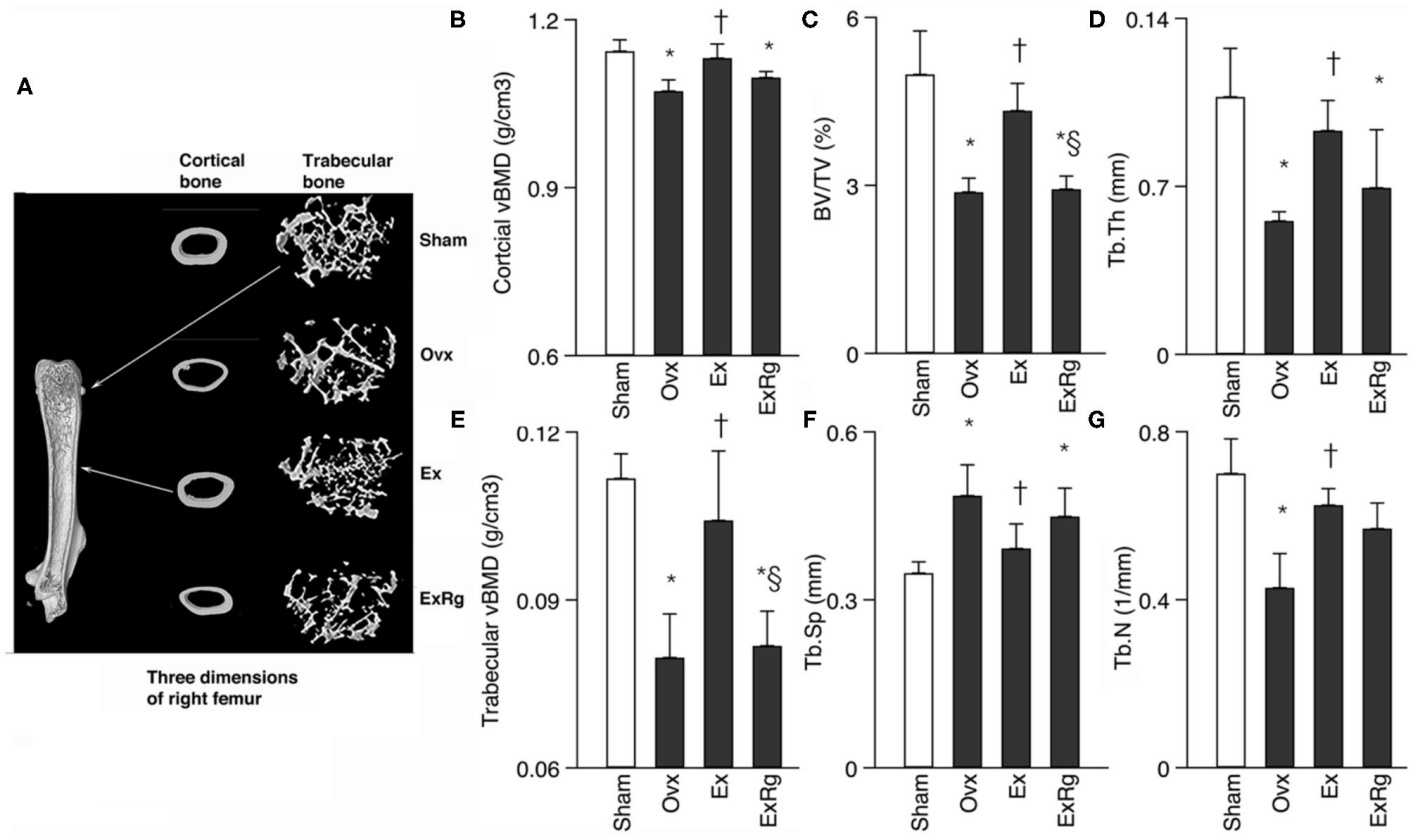
Comparisons between groups for cortical and trabecular volumetric bone mineral density (vBMD) and trabecular bone volume fraction (BV/TV), thickness (Tb.Th), numbers (Tb.N) and separation (Tb.Sp). Micro-CT analysis was performed for sham, ovariectomized (Ovx), Ovx plus exercise (Ex), and Ovx plus exercise and cRGDyk (ExRg) groups (each group, *n* = 10). Data are presented as mean ± SD. **(A)** Three dimensions of right femur; **(B)** cortical vBMD (g/cm^3^); **(C)** BV/TV (%); **(D)** Tb.Th (mm); **(E)** trabecular vBMD (g/cm^3^); **(F)** Tb.Sp (mm); **(G)** Tb.N (1/mm). *Denotes the difference between Sham group and Ovx, Ex, and ExRg groups was significant (*p* < 0.05); ^†^indicates the difference between Ovx group and Ex and ExRg groups was significant (*p* < 0.05); § presents the difference between Ex group and ExRg group was significant (*p* < 0.05).

Data of microCT measures showed that ovariectomy caused significant reduction of cortical and trabecular vBMD, trabecular BV/TV, Tb.Th and Tb.N, and enlargement of Tb.Sp (Figure 1). Eight-week exercise training could significantly rescue the decreased cortical and trabecular vBMD, trabecular BV/TV, Tb.Th and Tb.N, and reduce Tb.Sp. However, cyclo RGDyk treatment weakened the exercise-related improvement of trabecular vBMD and BV/TV (Figure 1).

### ALP Staining and Quantitative Analysis

**Figure 2:**
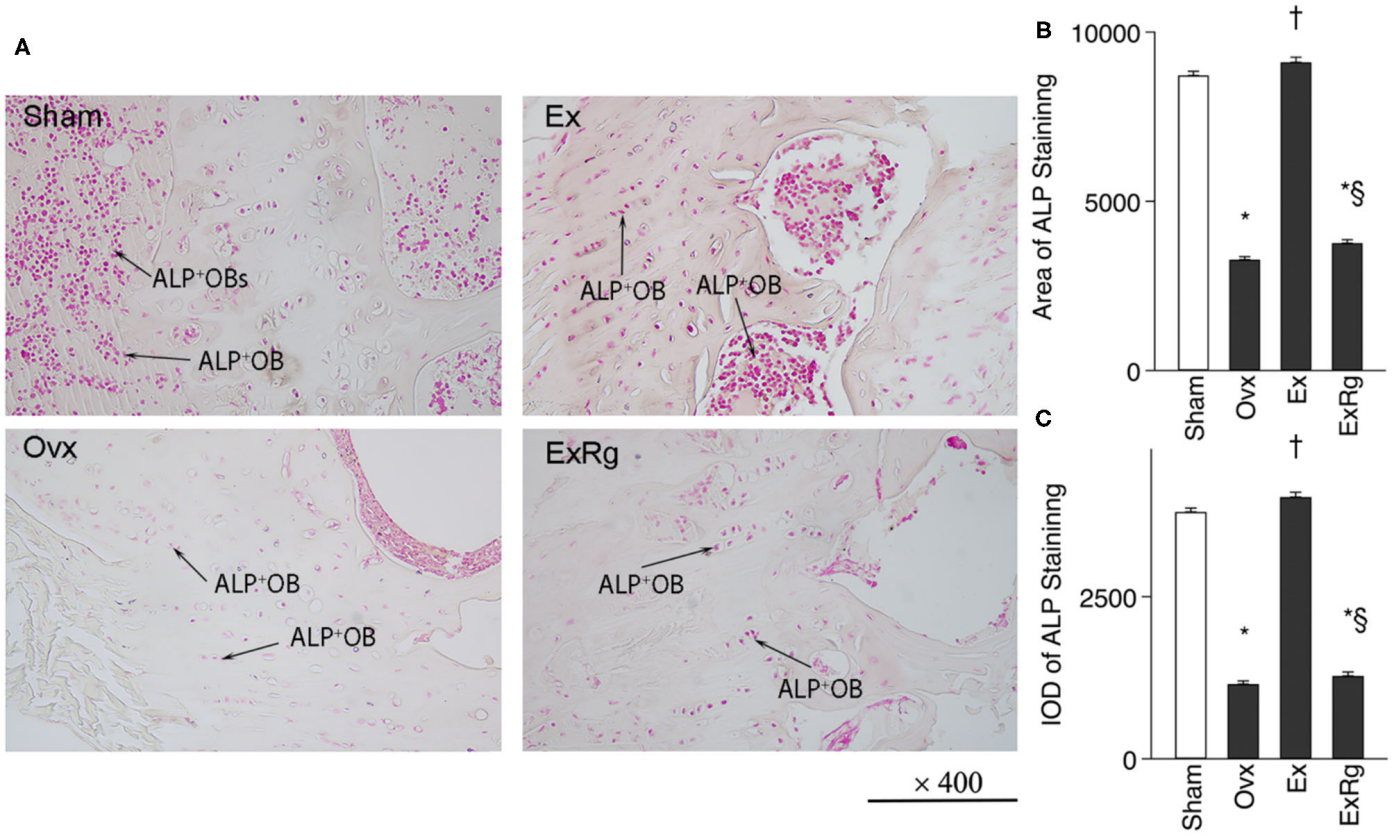
ALP staining and quantitative analysis. **(A)** ALP (alkaline phosphatase) staining was performed for sham, ovariectomized (Ovx), Ovx plus exercise (Ex), and Ovx plus exercise and cRGDyk (ExRg) groups (each group, *n* = 10). **(B)** ALP staining area. **(C)** ALP staining IOD (integral optical density). ALP^+^OB, ALP positive osteoblast. *The difference between Sham group and Ovx, Ex, and ExRg groups was significant (*p* < 0.05). ^†^ The difference between Ovx group and Ex and ExRg groups was significant (*p* < 0.05). ^§^The difference between Ex group and ExRg group was significant (*p* < 0.05).

Ovariectomy induced reduction of ALP^+^ cells in tibia compared with Sham operation, which were elevated by exercise intervention (Figure 2A). However, cyclo RGDyk treatment seemed to decrease exercise-induced increment of ALP^+^ cells. ALP staining quantitative analysis suggested that ALP intensity was lower in Ovx group compared with Sham group. Combined Ovx-exercise group had a higher ALP intensity in proximal tibia than the Ovx group, but the improvement could be diminished by cyclo RGDyk administration (Figures 2B,C).

### Western Blot and PCR Analyses

**Figure 3:**
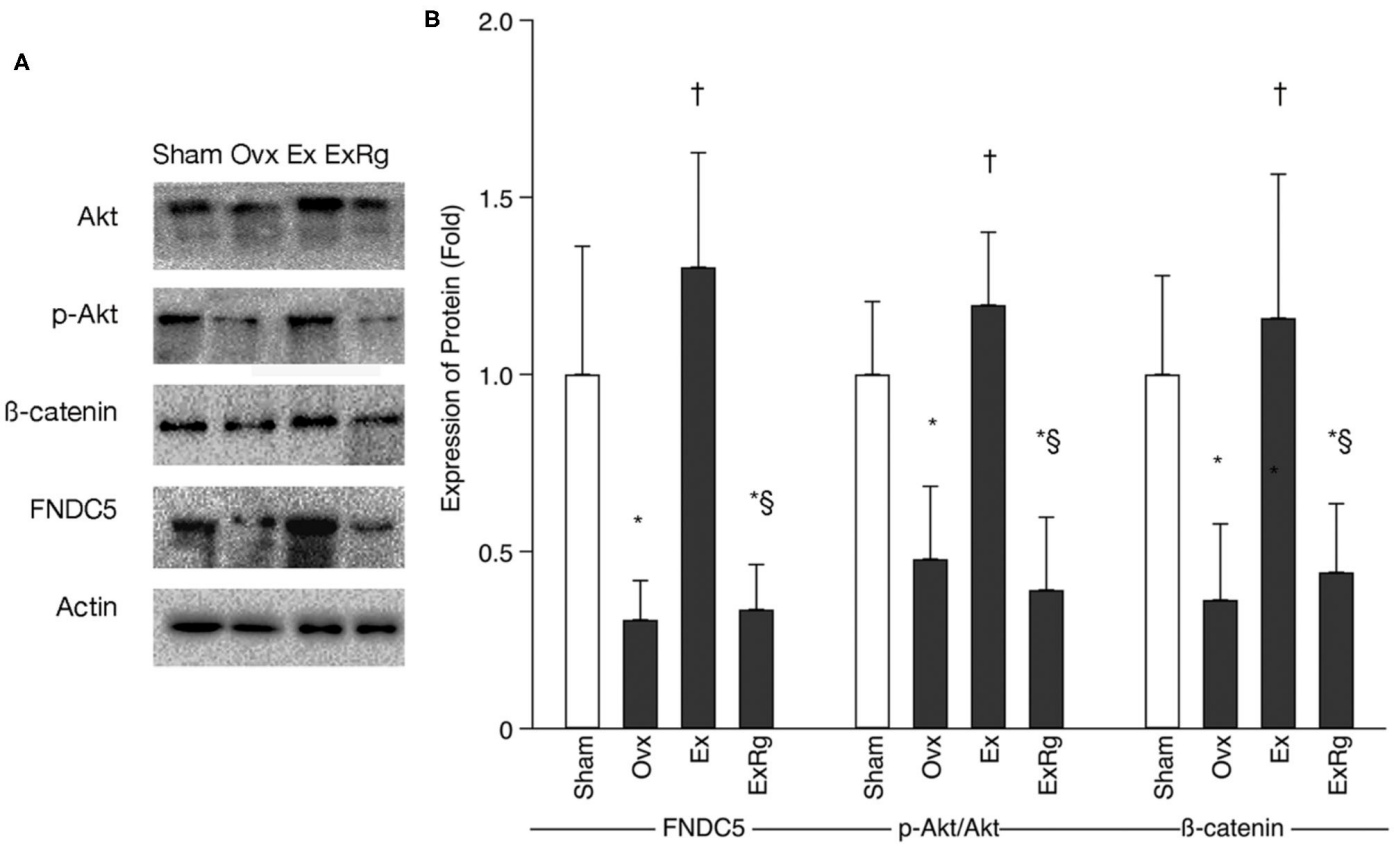
The protein expression of FNDC5, p-Akt/Akt, and β-catenin. **(A)** Western blot analysis for fibronectin type III domain-containing protein 5 (FNDC5), phosphorylated Akt (p-Akt), Akt, and β-catenin. **(B)** Expression of proteins (fold) for sham, ovariectomized (Ovx), Ovx plus exercise (Ex), and Ovx plus exercise and cRGDyk (ExRg) groups (each group, *n* = 10). Data are presented as mean ± SD. *Denotes the difference between Sham group and Ovx, Ex, and ExRg groups was significant (*p* < 0.05); ^†^indicates the difference between Ovx group and Ex and ExRg groups was significant (*p* < 0.05); § presents the difference between Ex group and ExRg group was significant (*p* < 0.05).

Western blot analysis indicated that FNDC5, the ratio of phosphorylated Akt (p-Akt) to Akt (p-Akt/Akt), and β-catenin levels were lower in Ovx group than Sham group (Figure 3). Exercise intervention up-regulated the protein expressions of FNDC5, p-Akt/Akt, and β-catenin. However, the exercise-elevated levels of FNDC5, p-Akt/Akt, and β-catenin decreased with cyclo RGDyk intervention (Figure 3).

**Figure 4:**
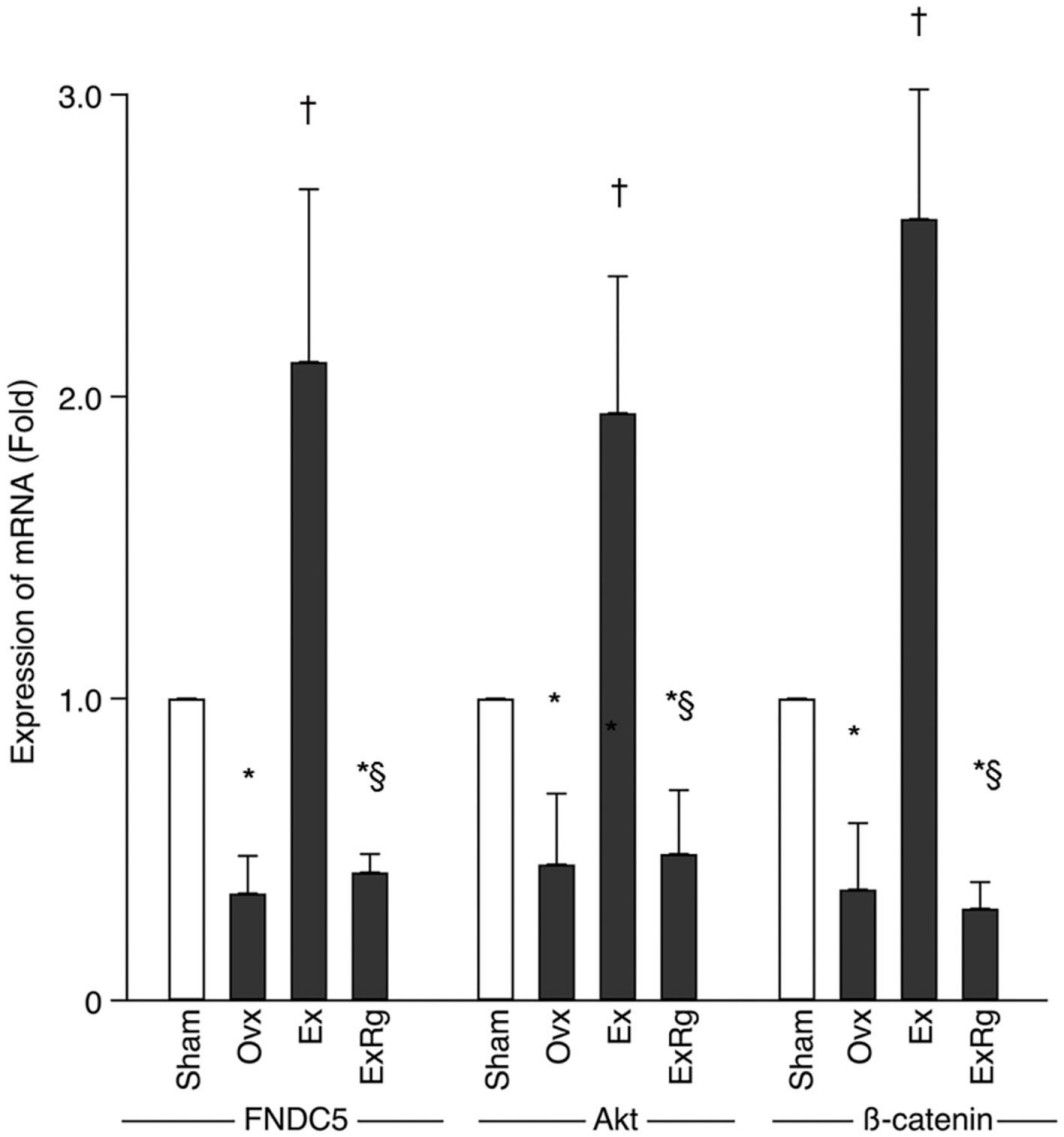
The mRNA levels of FNDC5, Akt, and β-catenin. PCR analysis was performed for sham, ovariectomized (Ovx), Ovx plus exercise (Ex), and Ovx plus exercise and cRGDyk (ExRg) groups (each group, *n* = 10). Data are presented as mean ± SD. FNDC5, fibronectin type III domain-containing protein 5. *Denotes the difference between Sham group and Ovx, Ex, and ExRg groups was significant (*p* < 0.05); ^†^indicates the difference between Ovx group and Ex and ExRg groups was significant (*p* < 0.05); § presents the difference between Ex group and ExRg group was significant (*p* < 0.05).

PCR analysis showed that mRNA levels of FNDC5, Akt, and β-catenin decreased in Ovx group compared with Sham group (Figure 4). Eight-week exercise training significantly improved the ovariectomy-associated reduction of FNDC5, Akt, and β-catenin mRNA. However, cyclo RGDyk administration blocked the increment of FNDC5, Akt, and β-catenin mRNA levels seen in Ex group (Figure 4).

## Discussion

Our findings proved exercise effective in preventing bone wasting induced by ovariectomy. Furthermore, the exercise-related improvement of bone mass could be attenuated by blocking of irisin receptor signalings, and Akt/β-catenin pathways might participate in this regulation process.

Isaksson and colleagues (Isaksson et al., 2009) reported that long-term exercise could generate beneficial outcomes on cortical and trabecular bone. Our findings were in agreement with previous reports. Recently, Luo and colleagues (Luo et al., 2020) found that recombinant irisin intervention could ameliorate bone loss in Ovx mice. Another study (Colaianni et al., 2017) also confirmed that irisin administration effectively prevented and restored bone loss in hind-limb suspended mice. Given the close correlation between exercise and irisin, it is suggested that irisin likely play an important role in regulating the effects of exercise on bone and blocking irisin pathways might affect skeletal response to exercise.

Kim and colleagues (Kim et al., 2018) first reported that by blocking irisin receptor (αV/β5), cyclo RGDyk could reduce irisin-induced signalings. Our findings indicated that cyclo RGDyk treatment decreased the exercise-related beneficial effects on cortical bone mass and trabecular BV/TV. Furthermore, exercise-elevated ALP staining positive osteoblasts were also affected by cyclo RGDyk. Colaianni and colleagues (Colaianni et al., 2015) reported that irisin treatment could promote osteoblast differentiation; and when irisin pathways were blocked, some osteoblastogenic genes were decreased (Kim et al., 2018), which might contribute to the cyclo RGDyk-induced reduction of osteogenic differentiation. Therefore, exercise-induced production of irisin helped to promote bone formation.

Several studies suggested that both endurance training (Miyamoto-Mikami et al., 2015; Korkmaz et al., 2019) and acute exercise (Nygaard et al., 2015; Kabak et al., 2018) could significantly rise circulating irisin concentrations. Our finding was in agreement with previous reports. Additionally, circulating BAP concentrations were up-regulated, whereas TRAP levels were down-regulated with exercise intervention, indicating that exercise had positive effects on bone metabolism. Given the important role of irisin in regulating osteoblastic differentiation (Qiao et al., 2016; Kim et al., 2018), the exercise-elevated irisin levels might increase its concentrations in bone tissue and then promote bone formation.

It is known that exercise is a strong stimulator for PGC-1α expression which in turn promotes FNDC5 expression in osteoblasts (Wrann et al., 2013) and subsequently regulates osteoblastic proliferation and differentiation (Qiao et al., 2016; Kim et al., 2018). Our studies agreed with previous findings, showing that exercise group had higher FNDC5, Akt and β-catenin mRNA levels in bone tissue, whereas the expression of Akt and β-catenin were down-regulated by blocking irisin signalings. Previous studies (Liu et al., 2015; Shi et al., 2017) reported that irisin regulated cell differentiations through promoting Akt/β-catenin pathway. Therefore, it was suggested that FNDC5/irisin signaling pathways affected the process of skeletal response to mechanical loading partly through its interaction with Akt/β-catenin pathway. Akt/β-catenin signaling pathway has been recognized as an important modulator in regulating the effects of mechanical strain on osteoblast differentiation (Sunters et al., 2010). The increased levels of Akt and β-catenin in osteoblasts can stimulate lymphoid-enhancing factor/T cell factor-mediated transcription (TCF/LEF) transcriptional activity of the osteopontin promoter, and then promote osteoblast differentiation (Armstrong et al., 2007; Sunters et al., 2010). However, because the interaction between irisin and Akt pathway is complicated and different results have been reported in previous studies (Liu et al., 2015; Shi et al., 2017; Zhang et al., 2019; Vadala et al., 2020), the findings should be interpreted with caution.

One limitation of this study is that since cyclo RGDyk is not a specific agent for blocking irisin signaling pathway, it might generate “non-specific” effects, for example, cyclo RGDyk also affects αvβ3 integrin signaling pathway (Yu et al., 2014) which possibly has effects on skeletal response to mechanical loading (Rubin et al., 2006). Several studies (Moghadasi and Siavashpour, 2013; Ketabipoor and Koushkie Jahromi, 2015) also reported that increased levels of estrogen were found after exercise intervention. The elevated circulating estrogen levels in exercise group implied that other pathways regulating skeletal response to exercise might exist. Armstrong and colleagues (Armstrong et al., 2007) reported that mechanical strain up-regulated estrogen receptor signaling pathways and then promoted osteoblast differentiation. This study did not conduct sham-operated exercise and sham-exercise plus cyclo RGDyk intervention groups which could help to detect the role of estrogen in affecting response of bone to exercise. It was another limitation. Given the fact that multiple molecular pathways involve in skeletal response to mechanical loading (Thompson et al., 2012), interactions between signaling pathways may exist. Therefore, to discern the specific effects of irisin interacting with other signaling pathways or the effects of cyclo RGDyk on irisin pathway, further studies are needed, e.g., adding sham-operated exercise and sham-exercise plus cyclo RGDyk intervention groups, and performing *in vitro* molecular experiment upon mechanical loading.

## Conclusion

Our findings have provided experimental evidence on the crosstalk between exercise and irisin in regulating skeletal response to endurance training. Irisin plays a potential role in regulating the beneficial effects of exercise on bone formation, partially through up-regulating Akt/β-catenin pathways. Future study should determine the molecular mechanism that irisin regulates osteoblast differentiation in response to mechanical strain.

## Data Availability Statement

The raw data supporting the conclusions of this article will be made available by the authors, without undue reservation.

## Ethics Statement

The animal study was reviewed and approved by The Animal Ethics Committee at University of Yangzhou.

## Author Contributions

RZ and WB designed the study and wrote the first draft of the manuscript. RZ, YZ, JLi, JLin, WC, YP, and WB performed the material preparation, data collection, and analysis. All authors read and approved the final manuscript.

## Conflict of Interest

The authors declare that the research was conducted in the absence of any commercial or financial relationships that could be construed as a potential conflict of interest.
